# First evidence of tick-borne encephalitis (TBE) outside of Hokkaido Island in Japan

**DOI:** 10.1080/22221751.2023.2278898

**Published:** 2023-11-14

**Authors:** Masayuki Ohira, Kentaro Yoshii, Yasuhiro Aso, Hideto Nakajima, Toru Yamashita, Ikuko Takahashi-Iwata, Norihisa Maeda, Katsuro Shindo, Toshihiko Suenaga, Tohru Matsuura, Kazuma Sugie, Tadanori Hamano, Akira Arai, Rikiya Furutani, Yasuhiro Suzuki, Chikako Kaneko, Yasuhiro Kobayashi, Eduardo Campos-Alberto, Lisa R. Harper, Juanita Edwards, Cody Bender, Andreas Pilz, Shuhei Ito, Frederick J. Angulo, Wilhelm Erber, Harish Madhava, Jennifer Moïsi, Luis Jodar, Hidehiro Mizusawa, Masaki Takao

**Affiliations:** a Department of Clinical Laboratory and Internal Medicine, National Center of Neurology and Psychiatry (NCNP), Tokyo, Japan; b National Research Center for the Control and Prevention of Infectious Diseases, Nagasaki University, Nagasaki City, Japan; c Department of Neurology, Oita Prefectural Hospital, Oita, Japan; d Department of Neurology, Nihon University Itabashi Hospital, Tokyo, Japan; e Department of Neurology, Okayama University Hospital, Okayama, Japan; f Department of Neurology, Hokkaido University Hospital, Hokkaido, Japan; g Department of Neurology, National Hospital Organization Beppu Medical Center, Oita, Japan; h Department or Neurology, Kurashiki Central Hospital, Okayama, Japan; i Department of Neurology, Tenri Hospital, Nara, Japan; j Division of Neurology, Jichi Medical University Hospital, Tochigi, Japan; k Department of Neurology, Nara Medical University Hospital, Nara, Japan; l Department of Neurology, University of Fukui Hospital, Fukui, Japan; m Aomori Prefectural Central Hospital, Aomori, Japan; n Department of Neurology, National Hospital Organization Shinshu Ueda Medical Center, Nagano, Japan; o Department of Neurology, National Hospital Organization Asahikawa Medical Center, Hokkaido, Japan; p Department of Neurology, Southern Tohoku General Hospital, Fukushima, Japan; q Vaccine Medical Affairs, Pfizer Japan Inc, Tokyo, Japan; r Medical Affairs, CSL Behring, Tokyo, Japan; s Vaccines, Antivirals, and Evidence Generation, Pfizer Vaccines, Collegeville, PA, USA; t Vaccines, Antivirals, and Evidence Generation, Pfizer Vaccines, Vienna, Austria; u Vaccines, Antivirals, and Evidence Generation, Pfizer Vaccines, London, UK; v Vaccines, Antivirals, and Evidence Generation, Pfizer Vaccines, Paris, France; w Department of Neurology, National Center of Neurology and Psychiatry (NCNP), Tokyo, Japan

**Keywords:** Tick-borne encephalitis, Far-Eastern subtype, arbovirus, flavivirus, tick-borne diseases, epidemiology

## Abstract

Tick-borne encephalitis (TBE) is an infection of the central nervous system caused by the tick-borne encephalitis virus (TBEV). TBE is endemic in parts of Europe and Asia. TBEV is transmitted to humans primarily by *Ixodes* ticks. There have been 5 TBE cases identified in Japan, all on the northern island of Hokkaido. Rodents with TBEV antibodies and *Ixodes* ticks have been identified throughout Japan, indicating that TBEV infection might be undiagnosed in Japan. Residual serum and cerebrospinal fluid (CSF) collected in 2010–2021 from 520 patients ≥1 year-of-age previously hospitalized with encephalitis or meningitis of unknown etiology at 15 hospitals (including 13 hospitals outside of Hokkaido) were screened by ELISA for TBEV IgG and IgM antibodies; TBEV infection was confirmed by the gold standard neutralization test. Residual serum was available from 331 (63.6%) patients and CSF from 430 (82.6%) patients; both serum and CSF were available from 189 (36.3%). Two patients were TBE cases: a female aged 61 years hospitalized for 104 days in Oita (2000 km south of Hokkaido) and a male aged 24 years hospitalized for 11 days in Tokyo (1200 km south of Hokkaido). Retrospective testing also identified a previous TBEV infection in a female aged 45 years hospitalized for 12 days in Okayama (1700 km south of Hokkaido). TBEV infection should be considered as a potential cause of encephalitis or meningitis in Japan. TBE cases are likely undiagnosed in Japan, including outside of Hokkaido, due to limited clinical awareness and lack of availability of TBE diagnostic tests.

## Introduction

Tick-borne encephalitis (TBE) is an infection of the central nervous system caused by a flavivirus, the tick-borne encephalitis virus (TBEV) resulting in symptoms of inflammation of the central nervous system (CNS) [1]. Three main TBEV subtypes have been described: European, Siberian, and Far-Eastern [1,2]; other TBEV variants have been identified with one of them, the Baikal subtype, recently proposed as a candidate for a fourth subtype [3]. TBE is endemic in northern, central, and eastern Europe and parts of Asia [1,2]. TBEV is transmitted to humans predominately by the bite of an *Ixodes* tick and, less commonly, by consumption of unpasteurized dairy products [2]. Most TBEV infections result in a febrile illness with fatigue, malaise, headache, and body pains [1,2]. Some infections have a biphasic course with an influenza-like illness, followed by a few days of well-being and then a second phase with neurological symptoms, including altered consciousness, ataxia, tremor, and nerve paralysis, and symptoms of CNS inflammation ranging from mild meningitis to severe encephalitis which can result in long-term neurological sequelae and death. Other TBEV infections result only in a monophasic neurological illness [1,2]. Monophasic TBEV infections are more common with infections of the Far-Eastern subtype [1]. Although supportive care is the mainstay of treatment for TBEV infection (i.e. there is no specific treatment for TBEV infection), early laboratory confirmation of TBEV infection is helpful for patient management by ruling out other causes of CNS inflammation.

Since 1993, there have been five TBE cases, resulting in two deaths, identified in Japan, all on the island of Hokkaido. The characterized TBEV strains causing these cases were of the Far-Eastern subtype [4-8]. Further evidence that TBEV, presumedly of the Far-Eastern subtype, may be endemic on Hokkaido is provided by detection of TBEV antibodies in rodents in Hokkaido and a 2010–2018 retrospective study that found undiagnosed TBEV infections in patients with neurological disorders on the island [9,10]. Although TBEV infections and TBE cases have not been reported outside of the island of Hokkaido, rodents with TBEV antibodies have been identified in Japan outside the island [11], and *Ixodes* ticks have been identified throughout Japan [12].

TBE is a nationally notifiable condition in Japan. Clinicians are required to report laboratory-identified TBEV infections in persons with symptoms of CNS inflammation (i.e. TBE cases) promptly to local public health centers [13,14]. According to the Japan Ministry of Health, Labour, and Welfare (MHLW), laboratory identification of a TBEV infection can be based on detection of TBEV antibodies by an enzyme-linked immunosorbent assay (ELISA) or a neutralization test (NT) [15]. Clinicians in Japan, however, seldom request TBEV diagnostics, apparently due to a low awareness of TBE [16]. If TBEV diagnostics are requested, the specimens must be sent to one of the few TBE reference laboratories in Japan because TBEV diagnostic kits, such as the commercial ELISA TBEV IgG and IgM antibody test kits, are not readily available. Additionally, the TBE NT uses TBEV, which is a bio-safety level 3 agent, requiring special laboratory handling that is only available at a reference laboratory [17]. At TBE reference laboratories, given the high specificity of NT, specimens from suspected TBEV-infected patients are commonly first screened by ELISA and then confirmed with the NT. Diagnosing TBEV infection using NTs is particularly important in Japan since interpretation of serologic test results can be hindered by cross-reactivity with Japanese Encephalitis virus [18,19]. Japanese Encephalitis is another flavivirus that is circulating in Japan and Japan’s National Immunization Program includes immunization with an inactivated Japanese Encephalitis virus. Furthermore, diagnosing TBEV infection using NTs is important in Japan because the ELISA diagnostic kits, which need to be imported from Europe, are produced using the European subtype of TBEV and these European ELISA test kits appear to be insensitive in detecting anti-TBEV antibodies produced in response to an infection with a TBEV of the Far-Eastern subtype, which is the most likely subtype to be present in Japan.

In addition to conducting public health surveillance for TBE, Japan conducts public health surveillance at sentinel medical facilities nationwide for cases of acute encephalitis. From 2007 to 2018, 5302 cases of acute encephalitis were reported to MHLW, of which 2633 (49.7%) were of unknown etiology [20].

We hypothesized that TBEV infections in patients with encephalitis or meningitis (i.e. TBE cases) have been missed in Japan since there are many cases of acute encephalitis and meningitis of unknown etiology in Japan, and there is a lack of commercially available TBEV diagnostics and an apparent lack of clinical awareness of TBE. Therefore, we retrospectively tested residual specimens collected from patients with encephalitis or meningitis of unknown etiology who were hospitalized throughout Japan to determine if TBEV infections and TBE cases were undiagnosed in Japan, particularly outside of Hokkaido.

## Materials and methods

**Figure 1.**
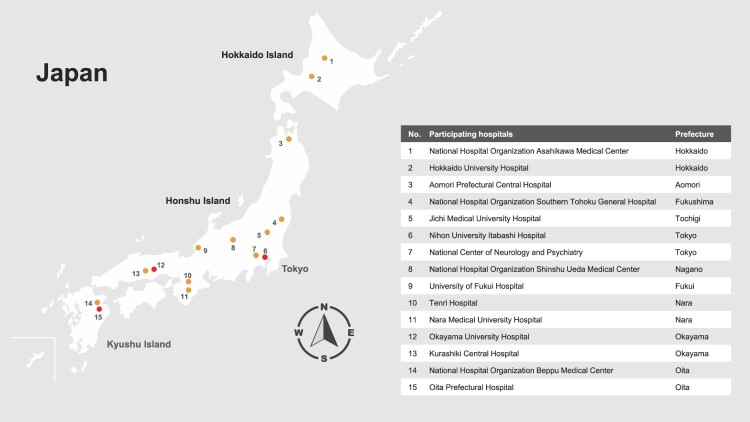
Location of participating hospitals, including those with tick-borne encephalitis virus infected case, by prefecture in Japan. Participating hospitals with a tick-borne encephalitis virus infected case are shown with a red circle.

Investigators at 15 hospitals (Figure 1), including 13 hospitals outside of Hokkaido, reviewed medical records to identify patients ≥1 year-of-age hospitalized from 2010 to 2021 with encephalitis or meningitis of unknown etiology for whom a standard-of-care serum and/or CSF specimen was collected, and to determine whether a residual amount of the collected serum or CSF specimen was stored at the hospital and was available for additional testing. The study, which was focused on testing residual specimens for evidence of infection with TBEV, Japanese encephalitis virus (JEV), or *Borrelia burgdorferi* sensu lato (*Bbsl*) was approved by the Medical Ethics Committee of the National Center of Neurology and Psychiatry (A2021-070), and by ethics review committees at Nagasaki University, Yamaguchi University, and each of the participating hospitals; testing residual specimens for other causes of encephalitis or meningitis was outside the scope of the study. Study information was posted on participating hospitals’ websites to provide patients an opportunity to decline participation. This manuscript focuses on the results of the TBEV and JEV testing; results of the *Bbsl* testing will be presented separately.

Investigators retrieved the stored residual sera and CSF specimens that had been collected from the previously hospitalized patients with encephalitis or meningitis of unknown etiology. Information on patient symptoms, medical and travel history, and disease outcomes was extracted from medical records using a structured study questionnaire. Residual specimens, after initial standard-of-care diagnostic testing at the time of patient’s hospitalization, had been stored in freezers at −20°C or below. Retrieved residual sera and CSF specimens were shipped overnight on dry ice at −80°C from participating hospitals to Nagasaki University by a commercial shipping company (Marken®, Durham, NC).

**Figure 2.**
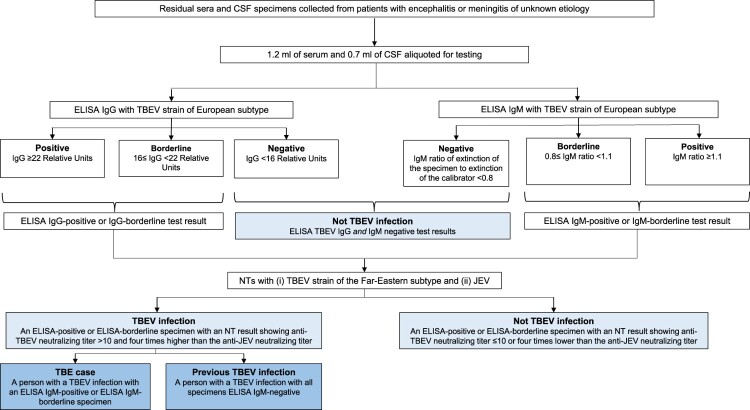
Case definitions for tick-borne encephalitis virus infection, tick-borne encephalitis case, and previous tick-borne encephalitis case, Japan, 2010–2021. CSF = cerebrospinal fluid; ELISA = enzyme-linked immunosorbent assay; IgG = immunoglobulin G; IgM = immunoglobulin M; JEV = Japanese encephalitis virus; NT = neutralization test; TBE = tick-borne encephalitis; TBEV = tick-borne encephalitis virus.

The residual specimens were defrosted at Nagasaki University, and 1.2 ml of serum and 0.7 ml of CSF were aliquoted and tested by ELISA for TBEV IgG and IgM antibodies using commercial test kits in accordance with the manufacturer’s instructions. The ELISA test kits were from EUROIMMUN® and are produced using the K23 TBEV strain of the European subtype (EUROIMMUN Medizinische Labordiagnostika AG, Lübeck, Germany). In accordance with the manufacturer’s guidelines, a specimen with an ELISA IgG ≥22 Relative Units was considered ELISA IgG-positive and ≥16 to <22 Relative Units was considered ELISA IgG-borderline (Figure 2). Following the manufacturer’s guidelines, a specimen with an ELISA IgM ratio of the extinction of the specimen to the extinction of the calibrator ≥1.1 was considered ELISA IgM-positive and ≥0.8 to <1.1 was considered ELISA IgM-borderline.

After being screened by ELISA for TBEV IgG and IgM antibodies, presence of a TBEV infection was confirmed by the testing of the ELISA-positive and ELISA-borderline specimens by NTs in a biosafety level three cabinet at the Nagasaki University laboratory according to standard procedures which have been previously described [9]. Since TBEV is known to serologically cross-react with other flaviviruses [18], ELISA-positive or borderline specimens were also tested by NT with JEV, the only other flavivirus endemic in Japan [19]. For the TBEV NT, a TBEV Oshima 5–10 strain that had been isolated from a patient with TBE in Hokkaido in 1993 [5] was incubated with serially diluted serum and inoculated to baby hamster kidney (BHK) cells. The cells were incubated with Eagle’s Minimal Essential Medium (FUJIFILM, Osaka, Japan) containing 1.5% carboxymethyl cellulose and 2% FBS for 4 days. After 4 days of incubation, the cells were fixed with 10% formalin and stained with 0.1% crystal violet. As NT was the confirmatory test in this study, a TBEV infection was defined as a NT-positive result which was an anti-TBEV neutralizing titer >10 that was at least four times higher than the anti-JEV neutralizing titer.

To distinguish between a current and a previous TBEV infection, we defined a current TBEV infection as a NT-identified TBEV infection in a patient with an ELISA IgM-positive (or borderline) serum specimen and a previous TBEV infection as a NT-identified TBEV infection in a patient with all specimens ELISA IgM-negative. Finally, in contrast to the European Centre for Disease Prevention and Control (ECDC) TBE case definitions which do not specifically include the NT [21], we used NT to confirm TBEV infection in our study and defined a TBE case as a current TBEV infection in a patient with symptoms of CNS inflammation.

## Results

A total of 761 residual specimens, collected from 520 hospitalized patients with encephalitis or meningitis of unknown etiology, were available for the study. The median (interquartile range) age of the 520 patients was 48 (31–68) years; 186 (35.8%) were aged ≥60 years. Specimens from 3 to 62 patients per hospital were available in the participating hospitals (Table 1). Of the 520 previously hospitalized patients, residual sera were available from 331 (63.7%) and residual CSF from 430 (82.7%) patients; both residual sera and residual CSF were available from 189 (36.3%) of the 520 previously hospitalized patients. No patients opted-out of the study.

**Table 1.** Tick-borne encephalitis virus infections among hospitalized patients with encephalitis or meningitis of unknown etiology with residual serum and residual cerebrospinal fluid specimen tested, by participating hospital, Japan, 2010–2021.

Hospital	No. hospitalized patients with encephalitis or meningitis of unknown etiology	No. (%) patients with residual sera available	No. (%) patients with residual CSF available	No. TBEV infections
National Hospital Organization Asahikawa Medical Center	34	0 (0)	34 (100)	0
Hokkaido University Hospital	60	53 (88.3)	22 (36.7)	0
Aomori Prefectural Central Hospital	62	53 (85.5)	58 (93.6)	0
Southern Tohoku General Hospital	5	4 (80.0)	2 (40.0)	0
Jichi Medical University Hospital	45	38 (84.4)	40 (88.9)	0
Nihon University Itabashi Hospital	39	4 (10.3)	35 (89.7)	1^a^
National Center of Neurology and Psychiatry	3	3 (100)	3 (100)	0
National Hospital Organization Shinshu Ueda Medical Center	25	3 (12.0)	25 (100)	0
University of Fukui Hospital	13	13 (100)	0 (0)	0
Tenri Hospital	10	7 (70.0)	7 (70.0)	0
Nara Medical University Hospital	30	13 (43.3)	24 (80.0)	0
Okayama University Hospital	53	47 (88.7)	52 (98.1)	1^b^
Kurashiki Central Hospital	46	32 (69.6)	39 (84.8)	0
National Hospital Organization Beppu Medical Center	60	40 (66.7)	54 (90.0)	0
Oita Prefectural Hospital	35	21 (60.0)	35 (100)	1^a^
TOTAL	520	331 (63.7)	430 (82.7)	3

^a^ TBE case.

^b^ Previous TBEV infection.

CSF = cerebrospinal fluid; TBE = tick-borne encephalitis; TBEV = tick-borne encephalitis virus.

TBEV infection was identified in 3 (0.6%) of 520 hospitalized patients with encephalitis or meningitis of unknown etiology (Table 2). Of the three TBEV-infected cases, two were TBE cases, and one was a previous TBEV infection.

**Table 2.** Enzyme-linked immunosorbent assay and neutralization test results for tick-borne encephalitis virus infected hospitalized patients with encephalitis or meningitis of unknown etiology at 15 participating hospitals in Japan, 2010–2021.

Age yrs. (sex)	ELISA (test result^a^)				NT		
	Serum		CSF				
	IgG	IgM	IgG	IgM	TBEV	JEV	Interpretation
61 (F)	2.90 (–)	0.98 (+/-)	1.09 (–)	0.07 (–)	40	<10	TBE case
24 (M)	3.20 (–)	1.46 (++)	NA	NA	160	<10	TBE case
45 (F)	94.73 (++)	0.45 (–)	0.16 (–)	0.07 (–)	160	<10	Previous TBEV infection

^a^ (–): negative; (+/-): borderline; (++): positive.

CSF = cerebrospinal fluid; ELISA = enzyme-linked immunosorbent assay; IgG = immunoglobulin G; IgM = immunoglobulin M; JEV = Japanese encephalitis virus; NA = not available; NT = neutralization test; TBE = tick-borne encephalitis; TBEV = tick-borne encephalitis virus.

### Case 1

Case 1 was a 61-year-old female with no known history of tick-borne diseases who had acute symptoms of CNS inflammation, including meningeal signs and nuchal rigidity. The patient was diagnosed as having acute encephalitis and was hospitalized at Oita Prefectural Hospital, on 6 November 2019. Onset of fever was recorded on 14 November 2019. The patient was hospitalized for 104 days. Upon discharge, the patient had marked residual neurological deficits. Prior to this study, the patient had not had a specimen tested for TBEV. In this study, residual serum collected on 6 November 2019 (the day of onset of illness and day of onset of neurological symptoms) was anti-TBEV ELISA IgG negative and IgM borderline. Residual CSF, collected on 15 January 2020 (eighty days after onset of illness), was anti-TBEV ELISA IgG- and IgM-negative but showed pleocytosis (>5 white blood cells per cubic millimeter). Residual serum collected on 6 November 2019, was NT tested and TBEV NT of 40 and JEV NT of <10. No other residual serum or CSF specimens were available. With a TBEV NT-positive specimen and anti-TBEV ELISA IgM borderline serum, the patient met the definition of an acute TBEV infection, and with the presence of symptoms of CNS inflammation, was considered a TBE case. Prior to illness onset, this patient lived in Bungotakada City which is on the southern island of Kyushu (Oita Prefecture) and >2000 km south of the island of Hokkaido. There was no record in her medical records of receipt of a TBE, yellow fever, or JE vaccine and no record of travel in the month prior to symptom onset.

### Case 2

Case 2 was a 24-year-old male with no known history of tick-borne disease who had fever onset on 2 February 2018. The patient was hospitalized at Nihon University Itabashi Hospital in Tokyo on 10 February 2018, when he showed symptoms of CNS inflammation which included nuchal rigidity. The patient was hospitalized for 11 days with full recovery at discharge. Prior to this study, the patient did not have a specimen tested for TBEV. In this study, residual serum collected on 10 February 2018 (eight days after onset of illness and on the day of onset of neurological symptoms) was anti-TBEV ELISA IgG negative and IgM positive. Residual serum was NT tested and had TBEV NT of 160 and JEV NT of <10. No other residual serum or CSF specimens were available. With a TBEV NT-positive and anti-TBEV ELISA IgM-positive serum, the patient met the definition of an acute TBEV infection, and with the presence of symptoms of CNS inflammation, was a TBE case. Prior to illness onset, the patient lived in Itabashi-ku, a ward of Tokyo, which is on the main island of Honshu and is >800 km south of the island of Hokkaido. There was no record in his medical records of receipt of a TBE, yellow fever, or JE vaccine and no record of travel in the month prior to symptom onset.

### Case 3

Case 3 was a 45-year-old female with no known history of tick-borne disease who had fever onset on 11 July 2018, and onset of altered consciousness on 14 July 2018. The patient was hospitalized on 15 July 2018, at Okayama University Hospital. She was hospitalized for 12 days with full recovery at discharge. Prior to this study, the patient had not had a specimen tested for TBEV. In this study, residual serum, collected on 16 July 2018, (five days after onset of illness and two days after onset of neurological symptoms) was anti-TBEV ELISA IgG positive and IgM negative; residual CSF collected on 16 July 2018, was anti-TBEV ELISA IgG and IgM negative. The residual CSF did not show pleocytosis. Residual serum was NT tested and had TBEV NT of 160 and JEV NT of <10. No other residual serum or CSF specimens were available. With a TBEV NT-positive and anti-TBEV ELISA IgG-positive serum, the patient met the definition of a previous TBEV infection. Prior to illness onset, the patient lived in Okayama (Okayama Prefecture) which is on the island of Honshu and is >1700 km south of the island of Hokkaido. There was no record in her medical records of receipt of a TBE, yellow fever, or JE vaccine.

## Discussion

Using the neutralization test, the gold standard diagnostic test for identifying TBEV infection, we identified three previously undiagnosed hospitalized TBEV-infected cases, which included two TBE cases, demonstrating that TBEV infections and TBE cases may go undiagnosed in Japan. Despite having symptoms of CNS inflammation (i.e. encephalitis and/or meningitis) of unknown etiology, none of the 520 hospitalized patients with encephalitis or meningitis in our study had been tested for TBEV prior to this study, illuminating the lack of TBEV diagnostic testing in Japan, presumedly due to limited clinical awareness and the absence of commercially available TBEV diagnostic tests. Each of the TBEV-infected cases was identified outside of Hokkaido. Furthermore, based on travel history of the two TBE cases, both cases apparently acquired the TBEV infection outside of Hokkaido. Combined with previous studies that identified TBEV antibodies in rodents outside of Hokkaido [11,16], results from our study indicate that TBEV transmission occurs in Japan outside of Hokkaido. Finally, the illness onset and specimen collection dates for the two TBE cases were in February and November. The purported vectors for TBEV transmission in Hokkaido are *Ixodes persulcatus* and *Ixodes ovatus* [16]. Both of these ticks are present nationwide and may therefore be a vector for TBEV transmission elsewhere in Japan [12]. Of note, tick bites in Japan occur primarily between April and September, particularly in May [12]. The significance of identifying TBE cases in patients with illness onset outside the summer months is not known and requires further study.

It is important to highlight the differences between the laboratory approach in our study and the laboratory approach used routinely by clinical laboratories in Europe for identifying a TBEV infection. The European Union (EU) TBE case definitions, used for reporting TBE cases to the ECDC, include detection of TBEV IgG and IgM antibodies in serum or CSF but do not specifically include NT [21]. ELISA is commonly used in clinical laboratories in Europe for TBEV diagnostics (i.e. to detect TBEV IgG and IgM antibodies in serum or CSF) due to technical simplicity of testing [22]. TBE ELISA test kits are particularly useful for clinical laboratories in Europe because there is limited circulation of other flaviviruses that can cross-react with TBEV. Furthermore, TBE ELISA testing can be conducted on the laboratory benchtop (biosafety level two) while NT requires a biosafety level three cabinet that is usually only available in a reference laboratory. The commercially-available TBE ELISA test kits in Europe, including the kits from EUROIMMUN which were used in this study, are based on the European TBEV subtype. The TBEV subtype most likely to cause infections in Japan, however, is the Far-Eastern subtype and the performance of ELISA TBEV test kits based on the European TBEV subtype against infections caused by the Far-Eastern TBEV subtype has not been well evaluated. Given the limitations of the available ELISA test kits for the diagnosis of TBEV infections in Japan, we used the more specific NT in our study using bio-safety level three laboratory procedures [17]. NT is generally considered the gold standard for serological diagnosis due to its high specificity [23]. Therefore, while the two TBE cases in our study do not meet the EU TBE case definition (which does not include NT), the cases in our study met a more specific study TBE case definition, using the gold standard NT, and are highly likely to represent acute TBEV infections (and hospitalized TBE cases) outside of Hokkaido in Japan.

In our study, the TBE NT, which utilized a TBEV strain of the Far-Eastern subtype, indicates that the TBEV infections were likely caused by the Far-Eastern subtype. The ELISA test kits used in our study did not detect IgG antibodies in either serum or CSF in either of the TBE cases. The residual sera for the two TBE cases were collected, respectively, on the day of illness onset and 8 days after illness onset. CSF was only collected from one of the two TBE cases and it was collected 80 days after illness onset. It may be that the ELISA test kits used in our study have a low sensitivity for detecting IgG in sera and in CSF in patients infected with the TBEV strain of the Far-Eastern subtype. If true, this supports the need to use the more sensitive and specific NT with the TBEV strain of the Far-Eastern subtype, rather than ELISA test kits based on the European subtype, for TBE diagnostics in Japan. The ELISA test kits used in our study were able to detect IgM antibodies in the serum of both TBE cases, although the TBE case that had serum collected on the day of illness onset had a borderline positive IgM test which may reflect the early collection of serum after illness onset.

Importantly, one of the TBE cases we identified had severe disease; the patient was hospitalized for >3 months and discharged with marked residual neurological deficits. Interestingly, this patient had monophasic disease with no reported symptoms prior to onset of symptoms of CNS inflammation. Such monophasic disease presentations are more common with TBEV infections of the Far-Eastern subtype than the European subtype [1].

A limitation of this retrospective study is that testing was contingent on the availability of residual specimens collected as a part of standard-of-care from previously hospitalized patients that had encephalitis or meningitis, and the specimens were stored for several years. Although at least one residual serum specimen and residual CSF specimen was available, respectively, from 63.7% to 82.7% of the previously hospitalized patients, both types of specimens were only available from 36.3% of the previously hospitalized patients. Additionally, the residual specimens were not always collected at the optimal time for detecting anti-TBEV IgG or IgM antibodies. For example, IgG and IgM antibodies are very commonly detected in the CSF >10 days after onset of neurological symptoms [24]. However, few of the residual CSF specimens in our study were collected more than 10 days after onset of neurological symptoms. Furthermore, as this was a retrospective study relying on residual specimens that were collected as part of standard-of-care, paired sera or paired CSF specimens from the same patient were not available so there was no mechanism for detecting increasing antibody levels. Had additional residual specimens been available or had the specimens been collected at more appropriate times for identifying an anti-TBEV antibody response, it is likely that additional TBEV-infected patients would have been identified. Another limitation, as this was a retrospective study utilizing information extracted from medical records of patients who were hospitalized with encephalitis or meningitis of unknown etiology, is that there was limited information available for each patient. For example, pertinent information in the medical record about patient symptoms was incomplete and, since there was a low clinical suspicion of TBEV infection for these patients, there was no information in the medical records about a history of tick bites or outdoor activities before symptom onset. Finally, this retrospective protocol did not include patient interviews as patients had been hospitalized between 2010 and 2021 and attempting to contact and interview the patients was beyond the scope of the study.

Based on the results of public health surveillance for acute encephalitis cases at sentinel medical facilities, MHLW estimates that there are 1600–1700 cases of acute encephalitis each year in Japan [20]. With a population of 125 million in 2022, this is an incidence of 1.4 acute encephalitis cases per 100,000 population per year, which is lower than the expected incidence of 6 per 100,000 population per year and may suggest an under-estimation of the number of acute encephalitis cases [25]. Of the reported acute encephalitis cases, almost half are of unknown etiology. Given the estimated number of cases of acute encephalitis each year in Japan and the fact that cases of meningitis are more common than cases of encephalitis, there are likely several hundred cases of acute encephalitis or meningitis of unknown etiology annually in Japan. Therefore, our finding that TBEV infection was the etiology for some patients hospitalized with encephalitis or meningitis of previously unknown etiology, suggests that TBEV infections and TBE may occur more commonly in Japan than previously thought.

## Conclusions

Using NTs, previously undiagnosed hospitalized TBEV-infected cases, including previously undiagnosed TBE cases, were identified in Japan outside of the island of Hokkaido. Results from this study demonstrate that TBEV infections and TBE cases are undiagnosed in Japan apparently due to lack of availability of TBE diagnostic tests. Taken together, our findings indicate that the burden of TBE in Japan is under-recognized, supporting the need for enhanced efforts to detect and prevent this serious disease. TBEV should be considered as a potential cause of encephalitis or meningitis in Japan, particularly in patients with encephalitis or meningitis of previously unknown etiology.

## Data Availability

The authors confirm that the data supporting the findings of this study are available within the article.
